# Airborne *Staphylococcus aureus* in different environments—a review

**DOI:** 10.1007/s11356-019-06557-1

**Published:** 2019-10-25

**Authors:** Anna Kozajda, Karolina Jeżak, Agnieszka Kapsa

**Affiliations:** Nofer Institute of Occupational Medicine, 8 Teresy Str, 91-348 Łódź, Poland

**Keywords:** *S. aureus*, MRSA, Bioaerosol, Indoor air, antibiotic resistance, Occupational exposure

## Abstract

The aim of the literature review was to describe the environments where the presence of airborne *Staphylococcus aureus* was confirmed and to catalogue the most often used methods and conditions of bioaerosol sampling to identify the bacteria. The basis for searching of studies on *S. aureus* in the bioaerosol in different environments was PubMed database resources from the years 1990–2019 (May). The review included studies which were carried on in selected environments: hospitals and other health care facilities, large-scale animal breeding, wastewater treatment plants, residential areas, educational institutions, and other public places. The highest concentrations and genetic diversity of identified *S. aureus* strains, including MRSA (methicillin-resistant *S. aureus*), have been shown in large-scale animal breeding. The role of the airborne transmission in dissemination of infection caused by these pathogens is empirically confirmed in environmental studies. Commonly available, well-described, and relatively inexpensive methods of sampling, identification, and subtyping guarantee a high reliability of results and allow to obtain fast and verifiable outcomes in environmental studies on air transmission routes of *S. aureus* strains.

## Introduction

Although the public health problem related to pathogenic bacteria of *Staphylococcus aureus* species is not new (Duckworth and Jordens 1990; Lowy 1998), yet in the last decade, year after year, more and more scientific alarms and reports about the increasing epidemiological risk have been coming from all continents (Agostino et al. 2017; Chang and Lin 2018; Denis 2017), especially in the context of acquiring by this species the genes of antibiotics resistance (Arias and Murray 2015; Epstein et al. 2016; Nadimpalli et al. 2018; Spellberg et al. 2008; Tacconelli et al. 2018). It has been estimated that complications in treatment refer, depending on the source, to 11–53% of *S. aure*us bacteremia (Keynan and Rubinstein 2013).

*S. aureus* is both a commensal (it colonizes nostrils, 20–40% of the general population are carriers) and a pathogen (causing mainly opportunistic infections of soft tissues, skin, and wounds, but also infections of blood, osteomyelitis, septic arthritis, endocarditis, pneumonia, and sepsis) (Gordon and Lowy 2008; Frank et al. 2010; Keynan and Rubinstein 2013; Nadimpalli et al. 2018; Otto 2010). *S. aureus* exhibits a number of properties which cause their high invasiveness (synthesis of enzymes which induce cytolytic effects, toxins which induce an inflammatory effect, and exotoxins causing the toxic shock syndrome and surface proteins, binding with cells, proteins, and blood cells in the attacked organism) (Gordon and Lowy 2008) and recurrent infections (formation of endospores and biofilms) (Conlon 2014). Furthermore, the *S. aureus* species is known for its easy acquisition of the genes of resistance to antibiotics (Chen 2013), especially from the group of β-lactams—methicillin-resistant *S. aureus* (MRSA) (Becker et al. 2014), but also to drugs from other groups of antibiotics (Long et al. 2006).

*S. aureus* exhibits many features which cause a high virulence and consequently also a high invasiveness (Gordon and Lowy 2008; Foster 2005). Considered as the most important virulence factors are the surface proteins described as microbial surface components recognizing adhesive matrix molecules, MSCRAMM (Foster and Hook 1998; Patti et al. 1994). These proteins bind the particles of collagen, fibronectin, and fibrinogen on the tissues of attacked organism (adherence to host tissue); therefore, they closely adhere to them and induce infections, including intravascular infections, those of bones, joints, or prosthetic-device infections (Gordon and Lowy 2008). Other factors of virulence comprise the formation of biofilms and endospores (Conlon 2014) and production of enzymes which induce the cytolytic effect, e.g. proteases, lipases, and evading or destroying of the host protection, e.g. leukocidins (Gordon and Lowy 2008; Foster 2005; Foster 2009). Other virulence factors include the following: cluster of genes called arginine catabolic mobile element (ACME), coagulase, or lactoferrine (Baba et al. 2002; Foster 2009; Gordon and Lowy 2008; Sollid et al. 2014).

Some strains of *S. aureus* are capable of producing specific immunomodulatory toxins which exhibit stimulating and mitogenic effects towards T lymphocytes, which may induce the collapse and toxic shock syndrome, including shock syndrome toxin-1 (TSST-1) (Sergelidis and Angelidis 2017). These toxins cause food poisoning (Denayer et al. 2017; Hennekinne et al. 2012; Sharma et al. 2018).

However, the most serious problem is that this species easily acquires resistance to many antibiotics simultaneously (multiantibiotics resistance), especially in the case of methicillin-resistant *S. aureus* (MRSA) (Becker et al. 2014; Boyce et al. 2005; Chen 2013; Gould et al. 2012; Paterson et al. 2014). According to the CDC report (2013), MRSA causes a number of diseases, starting from infections of skin and wounds to pneumonia and blood poisoning which may cause sepsis and death (Agostino et al. 2017; Daum 2007; David et al. 2014; Toro et al. 2014). Originally, MRSA constituted the main cause of nosocomial infections, but integrated preventive measures undertaken in health care facilities significantly decreased the percentage rate of nosocomial infections by as much as 54% during 2005–2011. It appeared, however, that the problem did not disappear but reached further than the hospital environment (hospital-associated MRSA, HA-MRSA), and in recent years, a continuous increase has been noted in MRSA infections in general population, especially the community-associated MRSA (CA-MRSA) and livestock-associated strains (LA-MRSA) (CDC 2013; Davis et al. 2012; Ferguson et al. 2016; WHO 2018). The epidemiological studies showed that the most common way of transmission of *S. aureus*, both sensitive and antibiotic-resistant strains, is the transfer type human-to-human through healthy animal farm workers (carriers of the bacteria) to their household members (Nadimpalli et al. 2018; Wardyn et al. 2015). The costs associated with treatment of the infection, prolonged hospitalization, and prevention of the spread of *S. aureus* are difficult to estimate, but they are surely very high (in billions of EUR annually) both for the health service, due to the undertaken procedures, and for the entire economy in relation to the absence from work and deaths (Antonanzas et al. 2015; Claus et al. 2014; Köck et al. 2010; Kraker et al. 2011; Lee et al. 2013).

Admittedly, the causes of the infection in humans are usually those strains which had colonized the anterior nares (DeLeo et al. 2010; Frank et al. 2010; von Eiff et al. 2001; Madsen et al. 2018a, b). Angen et al. (2017) showed in the experimental study with the volunteers that increased concentration of airborne MRSA could be an essential factor of the human nasal carriage of those strains. However, the authors conclude that a short-term inhalable exposure to MRSA can result in the nasal carriage, but young and healthy human organism is capable of removing pathogens within a few hours to a few days (Angen et al. 2017).

Therefore, the bacteria are particularly significant in the case of the people exposed environmentally, including hospitals and in such environments where there is a high probability of the occurrence of pathogenic and multiantibiotic-resistant strains, including the municipal wastewater treatment plants (WWTP) (Boopathy 2017; Gordon and Lowy 2008) or animal farms (Friese et al. 2013). The airborne transmission is as significant as the direct transmission of bacteria by the hands or mouth, although it may be difficult to indicate which of those routes is the most important for health care personnel (Bos et al. 2016; Chang and Lin 2018). *S. aureus* bacteria, similarly to other species from *Staphylococci* genus, are characterized by a high survival rate in dry environments, e.g. on surfaces or other materials commonly present at homes (Gupta et al. 2017; Kramer et al. 2006). Due to this feature, the settled dust can be a source of *S. aureus* and re-aerolisation of this dust causes an increased risk of bacteria inhalation (Madsen et al. 2018b). The studies prove that pathogenic strains of *S. aureus* are able to survive under unfavourable conditions, on dry surfaces, for a long period, even several months and still can be contagious for exposed humans (Beard-Pegler et al. 1988; Boyce 2007; Duckworth and Jordens 1990; Farrington et al. 1992; Hübner et al. 2011; Kramer et al. 2006). From this point of view, the most important factor associated with the presence and concentration of the *S. aureus* bacteria in the indoor air seems to be the air change rate (ACR), but also other factors such as relative humidity (RH) and season (Madsen et al. 2018b).

Methicillin-resistant *Staphylococcus aureus* (MRSA) occurs in public places and wastewater treatment plants, but it is most prevalent in hospitals (Agostino et al. 2017; Boopathy 2017; Boyce 2007; Boyce et al. 2005; Börjesson et al. 2010; Conceição et al. 2013; DeLeo et al. 2010; Otter and French 2009; Rosenberg Goldstein et al. 2012; Thompson et al. 2013; Wardyn et al. 2015). The direct sources of MRSA bacteria present in wastewater treatment plants are the wastes coming from hospitals and from the areas where animal husbandry is conducted (Wan and Chou 2014).

The studies carried out at homes confirmed the risk of exposure to airborne *S. aureus* in the residential environment (Gandara et al. 2006; Madsen et al. 2018b; Moon et al. 2014) and in public places (Conceição et al. 2013; Lenar-Boroń et al. 2014; Li et al. 2015; Lin et al. 2016; Messi et al. 2015; Moritz et al. 2015; Peng et al. 2015; Wlazło et al. 2008; Zhou and Wang 2013). Parrish et al. 2018 in the epidemiological study tried to find any relationship between the CA-MRSA soft tissue infections in children and their visits to public places within 3 months before hospitalization. However, the data analysis did not confirm any cause-and-effect relationship.

The aim of the literature review was to describe the types of environments where the presence of airborne *S. aureus* was confirmed and to catalogue the most often used methods and conditions of bioaerosol sampling to identify the bacteria.

## Material and methods

Investigations of *Staphylococcus aureus* bacteria presence in the bioaerosol in different environments were eligible for inclusion. The PubMed database was searched with crossing of the following keywords: ‘Staphylococcus aureus’, ‘MRSA’, ‘LA-MRSA’, ‘CA-MRSA’, ‘environment’, ‘environmental exposure’, ‘hospital’, ‘animal husbandry’, ‘CAFOs’, ‘WWTP’, ‘bioaerosol’, ‘indoor air’, and ‘air transmission route’ during 1990–2019 (May).

### Hospital environment

The hospital environment constitutes the main source and risk in spreading of *S. aureus* bacteria (CDC 2013). Interesting results were obtained by Frìaz-De León et al. (2016) who conducted in two hospitals of Mexico the research aimed at determination of the genera and incidence of airborne bacteria as well as the genetic similarity between environmental *Staphylococcus* spp. isolates (from the air) and clinical ones (from the patients). In the sampled air, the genus and species identification of isolated pure bacterial cultures was identified, and then, their sensitivity to antibiotics was analysed. It has been demonstrated that in the air of both hospitals, the bacteria of *Staphylococcus* genus dominated, while *S. aureus* took the fifth place of the total twenty five identified species. In the first hospital, 45% isolates belonging to *Staphylococcus* genus exhibited resistance to methicillin, but in the latter institution, none of the isolates was antibiotic resistant. The similarities between clinical and environmental *S. aureus* isolates were analysed using the dendrogram constructed according to the unweighted pair group method with arithmetic averages (UPGMA). The occurrence of three different genotypes was demonstrated. The first genotype is represented by the first group exhibiting 100% similarity, which comprises 6 clinical isolates of *S. aureus* coming from the patients, and this proves that they were infected with the same clone. The second genotype was identified in the clinical isolate coming from the patient hospitalized in the pulmonological ward, while the third genotype came from *Staphylococcus aureus* isolates occurring in the environment. These results undermined the hypothesis about the existence of a genetic relation between environmental and clinical strains, although the authors suspect that the probable cause was the too low quantity of analysed samples.

Beggs (2003) reviewed literature with the aim to confirm the role of airborne route in the transmission of nosocomial infections induced by MRSA strains and indicated that although there were strong foundations which in theory show that this route may be significant, yet this has not been confirmed empirically.

Mirzaei et al. (2014) conducted the research aimed at determination of the quantity and type of bacterial infections in hospitals. Seventy two air samples were collected from 3 operating rooms and from 3 consulting rooms, where 17 genera of bacteria, including *Staphylococcus* spp*.*, were found. Identification of the species showed that *S. aureus* at a concentration of 12.5 ± 7.5 CFU/m^3^ was dominant. Because of possible infections and prolonged wound healing time, the author of the studies suggests the need for monitoring of the concentration and species diversity of bioaerosols present in hospitals, especially in operating rooms.

Nandalal and Somashekar (2007) monitored for 2 years the incidence of *Staphylococcus aureus* bacteria in the hospital indoor air. The highest concentration of *S. aureus* bacteria, reaching 95 CFU/m^3^, was shown in the air sample collected in the paediatric ward. In the samples coming from the general ward, the concentrations of samples reaching 50, 63, and 75 CFU/m^3^ were found. In the air sample collected in the operating room, the value of 13 CFU/m^3^ was indicated; in the delivery room, it was 75 CFU/m^3^, in the endoscopy room 25 CFU/m^3^, and in the treatment room 63 CFU/m^3^. Simultaneously sampled was the outdoor air (control sample, outdoor background of the research), in which no *S. aureus* was identified. The authors concluded that because *S. aureus* occurred in all sampling places, it becomes necessary to monitor microflora in hospitals and undertake appropriate measures to prevent infections induced by airborne pathogens.

The studies on the quality of air and amount of bacteria and fungi in private and governmental public health care centres were conducted by Rangaswamy et al. (2013) with the aim to point out the level of airborne pathogens. The identified bacteria contained *S. aureus* species in different concentrations, depending on the air sampling place and time. In the case of samples collected in a private hospital, the highest concentration of *S. aureus* was identified in the emergency ward, in samples collected in the evening. As to the governmental health care centre, the highest *S. aureus* concentration – as in the case of a private health care centre – was identified in air samples collected in the evening and coming from the emergency ward. A high concentration was found in samples collected both in the morning and in the evening in the intensive therapy wards, pediatric and general wards. The authors indicate that because of the prevalence of airborne pathogenic *S. aureus* bacteria, the air quality has to be controlled.

Mandal et al. (2015) demonstrated that *S. aureus* bacteria, including antibiotic-resistant (also multiantibiotic-resistant) strains, were present in the wastewater discharged from hospitals. These wastes are supplied to the wastewater treatment plant, where they first constitute a source of exposure to those bacteria for workers and second become a potential threat to the environment. It was shown that in the treated water that is discharged into the environment (natural water reservoirs, soil), viable pathogens are still present, including *S. aureus* (Mandal et al. 2015).

### Concentrated animal feed operations

In the concentrated animal feed operation (CAFO), antibiotics are used preventively, before the first symptoms of the disease occur, which promotes spreading of antibiotic resistance (McCarthy et al. 2012; Love et al. 2011). Animal husbandry workers through their occupational contact become carriers, and consequently, bacteria with resistance genes may be conveyed to their families (Alvarado et al. 2009; Nadimpalli et al. 2018).

Ferguson et al. (2016) conducted the research on the identification of methicillin-resistant *S. aureus* (MRSA) in the indoor air of husbandry buildings and around them and also in the fodder prepared for the animals before they are brought to the breeding building and inside the building. Analysis of the size of viable particles was conducted using a 6-stage air sampler—Andersen. MRSA strains were found in all samples: in fodder and indoor air, they were present both in smaller particles and those bigger than 5 μm, whereas in atmospheric air at a distance of 215 m, they were found exclusively in particles smaller than 5 μm. The presence of MRSA in fodder before it was brought into the building points to a probability that fodder also can be a source of those bacteria. The mean concentration of MRSA on particles bigger than 5 μm in the samples from the pig house indoor air reached the value of 825 CFU/m^3^ and MRSA on the particles smaller than 5 μm reached 188 CFU/m^3^, whereas in outdoor air samples, it was 5 CFU/m^3^. For further research, 12 isolates of *Staphylococcus aureus* from the indoor air were analysed, 100% of which exhibited resistance to methicillin, 67% to tetracycline and clyndamycine, and 33% to erythromycin. Air sampling was also done after washing and disinfection of the pig house, and MRSA was not found there, which demonstrates that disinfection of interiors may be an effective form of preventing the airborne MRSA transmission.

To identify the sources of the origin of *S. aureus* in bioaerosol, inside 4 poultry farms, the samples of air and poultry faeces were collected; besides, the atmospheric air samples were collected at different distances from the buildings. The concentration of *S. aureus* bacteria inside the henhouses reached the values of 23, 27, 47, and 51 CFU/m^3^, considerably exceeding the concentrations identified in atmospheric air samples (1, 2, 4, 5, 10, 8, and 9 CFU/m^3^). Molecular methods applied in the research demonstrated a genetic similarity of isolated strains; 60% of *S. aureus* isolates present in the indoor air samples originated from manure samples. The results suggest that such pathogens as *S. aureus* while disseminating from manure to bioaerosol inside and outside the breeding facilities may constitute a potential source of infection (Zhong et al. 2009).

Liu et al. (2012) carried out some studies on the transmission of antibiotic-resistant genes of *S. aureus* strains in the environment. For this aim on 6 poultry farms, the samples of manure and indoor air in farming buildings and outdoor air were collected and analysed on the presence of *S. aureus* strains. Of 149 isolates with subtyped species, 15 types of antibiotics resistance were confirmed and 8 isolates exhibited resistance to methicillin. A high index of genetic probability at the level of 47.3–72.2% was shown between the isolates coming from the air, some of them exhibiting a 100% similarity to isolates sampled from manure. It has been demonstrated that multiantibiotic-resistant strains of pathogenic bacteria spreading in the air pose a risk to the health of the exposed people, thereby constituting a significant problem in public health.

Madsen et al. (2018a) conducted the study aimed at the measurement of the airborne MRSA and *S. aureus* particle sizes in the pig farms environment. The obtained results allow to conclude about the potential deposition of the bacteria in different parts of the exposed workers’ airways. The presence of the airborne *S. aureus* was identified in all 44 samples collected on the four farms using the six-stage Andersen sampler, and then, an analysis was made using *MALDI-TOF MS* method while MRSA was confirmed using the same methods in 31 of 33 samples collected on the three farms. The comparison of the geometric mean concentrations of *S. aureus* (1.8 × 10^3^ CFU/m^3^) and MRSA (447 CFU/m^3^) indicates that MRSA constitutes approx. 25% of the *S. aureus* concentration. The highest and lowest concentrations of the total inhalable MRSA were found in the empty stable during a high-pressure cleaning and in a stable with sick pigs, respectively. The highest and lowest concentrations of *S. aureus* were shown in a weaner stable and in feed storages, respectively. Analysis of the particle sizes indicated that most airborne *S. aureus* and MRSA strains were associated with particles between 7 and 12 μm and that the total inhalable MRSA and *S. aureus* concentration (100%) could be potentially deposited in the human respiratory system: in the upper airways 70%, in the primary and secondary bronchi 22%, and in the terminal bronchi and alveoli 8% (Madsen et al. 2018a).

### Wastewater treatment plant

Wastewater treatment plants may be a link in spreading of pathogenic bacteria to the environment. According to Berendonk et al. (2015), very high concentrations of bacteria, including pathogenic ones, and subtherapeutic concentrations of antibiotics present in wastewater treatment plants, result in introducing—to the environment—the antibiotic-resistant bacteria and genes resistant to antibiotics. Rosenberg Goldstein et al. (2012) analysed samples of wastewater collected in four wastewater treatment plants in the USA in view of the presence of those bacteria in all the investigated wastewater treatment plants in view of the occurrence of MRSA and MSSA strains. Those bacteria were found in all the investigated wastewater treatment plants; MRSA was present in 50% of the samples (24 of 44 samples), whereas MSSA was found in 55% (22 of 44) of the samples. Most of the MRSA strains and almost one third of MSSA (29%) exhibited resistance to two or more classes of antibiotics. According to the results of those studies, Kessler (2012) concludes that the confirmation of the presence of *S. aureus* bacteria in the wastewater of WWTPs explicitly indicates a high risk of exposure for WWTP workers and the people who live, work, or spend their time around more and more numerous agricultural and recreational areas irrigated with reclaimed wastewater. The presence of MRSA in the wastewater subjected to irrigation was indicated empirically (Börjesson et al. 2010). Moreover, Thompson et al. (2013) proved that MRSA strains present in wastewater originated from hospitals (HA-MRSA) can survive through all stages of treatment and are present in reclaimed wastewater. The same opinion as regards bacteria and genes of resistance present in wastewater was expressed by Baquero et al. (2008)*.* Apart from hospitals, an important source of antibiotic-resistant *S. aureus* strains is concentrated animal feed operations (Harper et al. 2010). Wan and Chou (2014) analysed wastewater coming from slaughter houses of pigs in view of the prevalence of LA-MRSA and *mecA* genes (the gene coding resistance to methicillin) and showed the presence of both agents in high concentrations.

The wastewater treatment processes generate high amounts of bioaerosol. This is mainly due to the processes of mixing, overflowing, and aeration necessary to the biological treatment, which, however, cause splashing, bubbling, and spraying (Fracchia et al. 2006). The respiratory tract can be a significant route of infection for WWTP employees (Bos et al. 2016). Protecting of the respiratory tract from exposure to high concentrations of microorganisms still is a challenge in a field of occupational safety and hygiene. Although in developed countries, the masks with biological filters are commonly available and used in practice, yet it was shown empirically that such masks had some defects. Majchrzycka et al. (2016) tested the survivability of certain species of microorganisms on the filtration fabric from masks protecting the respiratory tract and showed that it was the *S. aureus* species that with available 40–200% humidity exhibited the highest survivability.

Fracchia et al. (2006) conducted the research aimed at assessment of the level of air pollution with bacterial microflora (including pathogenic) in two wastewater treatment plants with different treatment technologies in Italy. It was demonstrated that the *S. aureus* species was present in the air both in summer and winter season, but no correlation was found between the season and bacteria airborne concentration. The results confirm an increased risk of infection in wastewater treatment plant workers and point to the need for rigorous use of preventive measures. Much more frequently, the research conducted in the wastewater treatment plant environments related to the identification of airborne bacteria only to the level of *Staphylococcus* genus (De Luca et al. 2001; Gotkowska-Płachta et al. 2013; Han et al. 2018; Uhrbrand et al. 2017).

Michałkiewicz et al. (2011) analysed the microflora present in atmospheric air in different sites of the wastewater treatment plant in different seasons in Poland and confirmed the presence of, among other, *S. aureus* species in bioaerosol. Vantarakis et al. (2016) tested also the qualitative composition of bacterial microflora in immediate vicinity of the municipal wastewater treatment plant (500 m). For species identification, randomly selected were 83 bacterial isolates among which 29% (24 isolates) were those of *S. aureus* genus.

### Public places and homes

Li et al. (2015) conducted a study aimed at the confirmation of the presence of *S. aureus* bacteria, including MRSA strains, in indoor air of various public buildings in China in spring, analysing inhalable and respirable fractions. For both analysed fractions of bioaerosol, the highest concentration of *S. aureus*, presented as the median (84 CFU/m^3^ for inhalable fraction and 57 CFU/m^3^ for respirable fraction), and also MRSA strains (33 CFU/m^3^ for inhalable fraction and 18 CFU/m^3^ for respirable fraction), was identified at the cinema hall. Moon et al. (2014) conducted the studies aimed mainly at the identification of microorganisms isolated from the air inside the flats in South Korea. The predominant bacteria were *Staphylococcus* (which constituted 49–61.3% of all microfloras). The presence of *S. aureus* species was confirmed in all investigated apartments in 4–140 CFU/m^3^ concentrations. MRSA was isolated from air samples from 66% of apartments covered by the measurements.

Messi et al. (2015) conducted the research in different environments, including public places in Italy, related to the identification of bacteria exhibiting antibiotics resistance. Air samples were collected from restaurants, fitness clubs, offices, houses, and classrooms, as well as operating rooms, dentists’ surgeries, and waste management plants. Among 280 analysed isolates, 17% were classified to *Staphylococcus* genus, within which the presence of five *S. aureus* isolates belonging to two different strains was found. Two *S. aureus* isolates exhibited resistance to erythromycin and tetracycline. Pastuszka et al. (2000) analysed the concentration of microorganisms in indoor air of flats. Samples of bioaerosol in the flats were collected in summer and winter seasons; the atmospheric air constituted the outdoor background of the study. In the identified microflora was a present *S. aureus* strain (7.8% of the total bacterial microflora). A similar study was conducted by Gandara et al. (2006) who in Texas isolated from the flats’ indoor air the *S. aureus* bacteria in average concentration of 15 CFU/m^3^, whereas in 23 samples collected outdoors, the mean concentration of *S. aureus* reached 12 CFU/m^3^. The analysis of antibiotic resistance showed the presence of *S. aureus* strains resistant to penicillin and ampicillin (in 22 flats) and to cefaclor (in 14 flats).

The studies demonstrate that *Staphylococcus* spp. bacteria resistant to various groups of antibiotics are present in public transport means, and passengers’ migration at transfer stations provides conducive conditions to the dissemination of antibiotic-resistant strains (Lin et al. 2016; Peng et al. 2015; Zhou and Wang 2013), including also MRSA (Conceição et al. 2013).

Wlazło et al. (2008) conducted in Poland the study aimed at the evaluation of library staff’s exposure to biological hazards. In the samples collected from the bioaerosol and dust settled on book surfaces, 53 species of bacteria belonging to 20 genera were identified. The qualitative analysis of the bacteria of *Staphylococcus* genus showed the presence of 14 species, including *S. aureus*. The studies on microbiological quality of air in 8 libraries of Ethiopia were conducted by Hayleeyesus and Manaye (2014). In total, 96 air samples were collected and microorganisms were identified, among which *S. aureus* was isolated.

Also in Poland, Lenar-Boroń et al. (2014) carried out a similar study, but they collected the air samples at a students’ hostel. In the collected bioaerosol, *Staphylococcus* bacteria were isolated and identified to the species, and subsequently, their antibiotics resistance was analysed. Of the total of 5 species of staphylococci, *S. aureus* resistant to erythromycin and clindamycine was isolated.

### Methodological catalogue

According to the literature (Agersø et al. 2013; Madsen et al. 2019), the correct option from the variety of methods of air sampling for microbiological analysis requires the huge knowledge and long experience in the field study. Many different factors can influence the result, such as the concentration of airborne microorganisms (closely related with the type of environment), relative humidity (RH), temperature, species composition, and their ability to survive under unfavourable conditions. Therefore, it is important to select not only the sampling method and the type of the air sampler, but also the appropriate sampling conditions (the most important are time, air volume, and air flow rate). The next critical step is the selection of the type of medium (e.g. enriched or selective and differential media), cultivation conditions, and identification or molecular typing. The laboratory recommendations related to the microbiological analysis of *S. aureus* including MRSA strains are described in detail in literature and are updating according to the current state of the knowledge (Deplano et al. 2018; Missiakas and Schneewind 2013; Vitko and Richardson 2013).

According to the literature, a modern and expensive equipment is not always better than the traditional methods of sampling. Smith et al. (2018) in a study conducted to assess the cleanliness of a hospital intensive care unit analysed samples from surfaces and from the air (sampled by passive and active methods) on the presence of *S. aureus* and MRSA. The results obtained from surfaces and air were compared, and their analysis indicates that the sedimentation method is in line with the results of the surface study. The authors concluded that the settle plates could be applied for routine environmental screening to determine the infection risk in intensive care units.

However, if there is a necessity to confirm species or strains, the MALDI-TOF MS method (matrix-assisted laser-desorption/ionization time-of-flight mass spectrometry) or genetic methods have eliminated the risk of erroneous identification. Currently, the trend in the use of these two methods of microorganism identification has changed; MALDI-TOF has become a standard in both clinical and scientific research, in which genetic methods have been more prominent until recently. Due to the inconsistencies of the genetic techniques, i.e. high price, necessity of participation of specialized personnel, and relatively high time-consuming procedure, more and more laboratories use microbial identification by MALDI-TOF. The advantages of MALDI-TOF MS involve simplicity of sample preparation, relatively low costs, and fast analysis with high reliability of results comparable with the subtyping. Owing to these assets, the MALDI-TOF MS has become a standard in microbiology (Kostrzewa and Nagy 2016).

Madsen et al. (2019) conducted the study which can be a basis for the description of relevant differences in results (understood as the concentration of airborne MRSA) between six methods of sampling of airborne MRSA (five active and one passive) on five pig farms. Identification of bacteria was carried out on selective agar media using the MALDI-TOF MS method. The study showed a variation in bacteria concentrations between different methods of sampling. Geometric mean concentrations were in the range of 1450–2000 CFU/m^3^ and 110–419 CFU/m^3^, for S. *aureus* and MRSA, respectively. The authors tested also the active sampling methods for the half-life of bacteria, and the survival of *S. aureus* and MRSA was studied for day 0–day 6 after sampling. The study showed no significant effect of the active sampling method during 1 h on the half-life of studied pathogens, but the passive sampling should not exceed 3 days due to overloading and the die-off of MRSA*.* The authors found a significant correlation between the dust and *S. aureus* concentration within farm section and different farms but not in all the investigated facilities. The results showed a high spatial and temporal variation of bacteria concentrations; therefore, the authors concluded that to obtain a reliable picture of a potential exposure, samples should be taken repeatedly and in different areas within a stable section.

On the basis of the literature selected for the review, the methods of sampling and identification or subtyping of *S. aureus* including MRSA strains were catalogued and presented in Table 1.

**Table 1:** Catalogue of sampling and analysis methods applied in environmental studies on exposure to *S. aureus* based on literature

Lp.	Author	Environment	Sampling method/equipment1	Sampling time2	Air flow3	Medium	Identification/subtyping method
1	Sattar et al. (2016)	Aerobiology chamber	Air sampler: Particle Measuring Systems, Boulder	2 min	28.3 L/min	Streptomycin thallous acetale (STA)	Morphology
2	Ferguson et al. (2016)	Inside and downwind of a swine facility	6–STG Andersen air sampler	n/d	28.3 L/min	CHROMagar™Staph aureus	Molecular tests
3	Mirzaei et al. (2014)	Surgery rooms and emergency departments	1–STG Andersen air sampler and SIBATA air pump	10 min	28.1 L/min	Blood agar and brain heart infusion agar (BHIA)	Biochemical tests
4	Hayleeyesus and Manaye (2014)	Jimma University libraries	Sedimentation	30, 60, 90 min	n/d	2% nutrient agar	Standard methods
6	Chang and Wang (2015)	Biological safety cabinet placed in a biosafety level II laboratory	Air samplers: AGI–30, BioSampler, and 1–STG Andersen air sampler	3, 6, 15, 30, 60 min	12.5 L/min	Tryptic soy agar (TSA), mannitol salt agar (MSA)	n/d
7	Moon et al. (2014)	Residential apartments	Air impactor: MAS–100, Merck	2 min	100 L/min	CHROMagar™Staph aureus, CHROMagar™MRSA	Morphology
8	Liu et al. (2012)	6 chicken farms	6–STG Andersen air sampler	1–5 min	28.3 L/min	Baird–Park agarose	Biochemical tests
9	Wu et al. (2011)	Nursing care institution	Air sampler: Airport MD8, Sartorius	5 min	50 L/min	Gelatin membranes	Molecular methods
10	Zhong et al. (2009)	Chicken house	6–STG Andersen air sampler	1–5 min	28.3 L/min	Baird–Park agarose	Biochemical tests
11	Alvarado et al. (2009)	Dairy cat	two-stage ambient culturable sampler system connected to a pressure/vacuum pump	30, 60, 120 s	28.3 L/min	Tryptic soy agar (TSA)	Biochemical tests
12	Nandalal and Somashekar (2007)	Hospital	Handhold Reuter air sampler	n/d	n/d	Mannitol salt agar (MSA)	Biochemical tests
13	Gandara et al. (2006)	24 one-storey houses that had no basement or attic	2–STG Andersen air sampler	10, 15 min	28.3 L/min	Tryptic soy agar (TSA)	Biochemical tests
14	Frìaz-De León et al. (2016)	Two health institutions	M Air T air sampler	Less than 7 min	140 L/min for the first, 500 L/min, then 180 L/min	Blood agar	Biochemical tests
15	Kumar and Goel (2016)	14 residential houses	Reuter Centrifugal Sampler	2 min	280 L/min	Mannitol salt agar (MSA), and mannitol salt agar (MSA) containing methicillin	Biochemical tests, molecular methods
16	Messi et al. (2015)	Indoor environments: gyms, restaurants, homes, offices, classrooms, public and private clinics, operating rooms, etc.	Portable air microbiological sample, surface air system (SAS)	n/d	n/d	Plate count agar (PCA)	Automatic biochemical test
17	Saadoun et al. (2014)	Hospital	Microbiological air sampler: M.AQS II-90, Oxoid	2 min	100 L/min	Tryptic soy agar (TSA)	Biochemical test
18	Li et al. (2015)	General hospital, kindergarten, hotel, movie theatres, university teaching building	6–STG Andersen air sampler	8–12 min	28.3 L/min	Baird–Parker agar	Biochemical tests
19	Rangaswamy et al. (2013)	A private and a government tertiary health care centre	Merck Microbial Air Sampler MAS–100NT	30 s	100 L/min	Soyabean casein digest agar (SCDA)	Morphology and biochemical tests
20	Stryjakowska-Sekulska et al. (2007)	University rooms	Sedimentation	15 min	n/d	Yeast extract agar (YEA)	Biochemical tests
21	Pastuszka et al. (2000)	Homes and office rooms	6–STG Andersen air sampler	5–10 min	n/d	Tryptic soy agar (TSA)	Biochemical tests
22	Wlazło et al. (2008)	17 libraries	6–STG Andersen air sampler	5 min	n/d	Tryptic soy agar (TSA)	Biochemical tests
23	Lenar-Boroń et al. (2014)	Dormitory	MAS–100 Air Sampler, Merck	n/d	n/d	Chapman media	Morphology
26	Skóra et al. (2012)	Museum	MAS–100 Eco Air Sampler, Merck	n/d	n/d	Tryptic soy agar (TSA)	Biochemical tests
27	Korzeniewska et al. (2008)	Wastewater treatment plants	Sedimentation	n/d	n/d	Chapman media	Biochemical tests
28	Fracchia et al. (2006)	Wastewater treatment plants	Agar impact sampler surface air system (SAS)	20 s	180 L/min	Plate count agar (PCA)	Biochemical tests
29	Michałkiewicz et al. (2011)	Around municipal wastewater treatment plants	Two methods: sedimentation and aspiration	10 min (sedimentation method)	n/d	Chapman medium	Biochemical tests
30	Vantarakis et al. (2016)	Around wastewater treatment plants	Air sampler: International PBI Surface Air System, SAS	15 min	90 L/min	Tryptic soy agar (TSA)	Biochemical tests
31	Feld et al. (2018)	Swine farms	Electrostatic dust fall collectors: EDCs	n/d	n/d	Brilliance MRSA 2 agar (chromogenic medium for MRSA)	Molecular method
32	Masclaux et al. (2013)	Pig farms	MAS–100 Eco Air Sampler, Merck	6 min	100 L/min	ChromoID *S. aureus*	Molecular method
33	Zhou and Wang (2013)	Six different interchange metro station, six different hospitals, and the grassland of two parks	Sedimentation	5 min	n/d	Nutrient agar	Biochemical tests
34	Kooken et al. (2012)	Occupied and unoccupied rooms in a suburban elementary school in Columbia	N6 Single Stage, Viable Impactor	n/d	28.3 L/min	Sheep blood agar (SBA)	Molecular tests
35	Torres et al. (2013)	Cafeteria	Staplex MBS-6; 6 Stage Microbial Air Sampler	30 min	n/d	Brain heart infusion agar (BHIA)	Molecular tests
36	Karwowska (2005)	Barns (cow sheds, pigsties, poultry houses) and dairy buildings	MAS–100 Air Sampler, Merck	0.5–2.5 min	100 L/min	Chapman agar	Biochemical tests
37	Kubera et al. (2015)	Public kindergarten and nonpublic kindergarten	Sedimentation and MAS–100 Air Sampler, Merck	30 min (sedimentation method)	n/d	Mannitol salt agar (MSA)	Morphology
38	Madsen et al. (2018b)	Farm homes; homes and offices in the city area	Gesamtstaubprobenahme sampler (GSP)	1 – 5 h	3.5 L/min	Nutrient agar	MALDI-TOF MS (matrix-assisted laser desorption/ionization time-of-flight mass spectrometry); molecular method
			Electrostatic dust collectors (EDCs)	12–16 days, 1 month	n/d	Nutrient agar	
			Exhaled breath condensate	4 min	42 μL_EBC_/min	SaSelect agar (chromogenic medium for *S. aureus*)	
39	Madsen et al. (2019)	Pig farms	Electrostatic dust collectors (EDCs)	60 min	n/d	Brilliance MRSA 2 agar (chromogenic medium for MRSA), SaSelect agar (chromogenic medium for *S. aureus*)	MALDI-TOF MS
			Gesamtstaubprobenahme sampler (GSP)	60 min, 120 min	12.5 L/min		
			Andersen six-stage cascade impactor (ASCI)	10 min	28.3 L/min		
			Institute of Occupational Medicine air sampler (IOM)	120 min	12.5 L/min		
			Impinger	120 min	12.5 L/min		
			Triplex cyclone	120 min	12.5 L/min		
40	Madsen et al. (2018a)	Pig farms	Andersen six-stage cascade impactor (ASCI)	2–40 min	28.3 L/min	Brilliance MRSA 2 agar, SaSelect agar	MALDI-TOF MS
			Gravimetric impactor DGI-1570	15 min	50 L/min	Brilliance MRSA 2 agar, SaSelect agar	
			Respicon particle sampler	3 h	3.1 L/min	Brilliance MRSA 2 agar, SaSelect agar	
41	Smith et al. (2018)	Intensive care unit in a hospital	Double-sided dipslides method	10 s	n/d	Nutrient agar, staphylococcal selective agar, *S. aureus* identification (SAID) agar	Automated susceptibility testing (Vitek)
			Sedimentation method	1 h	n/d		
			MAS–100 Air Sampler, Merck	1 min	116 L/min		

^1^Manufacturer’s name of samplers

^2^*h* hours, *min* minutes, *s* seconds

^3^*L/m* litres per minute

*n/d* no data (lack of information in the methodology)

## Conclusions

The literature review points to a common presence of airborne *S. aureus* bacteria in different environments. In the air was confirmed also presence of multiantibiotics-resistant strains, including MRSA. The presented results of the research demonstrate that a particular risk for public health, apart from hospitals, refers to the environment of wastewater treatment plants and livestock husbandry, where the high concentrations of *S. aureus*, including antibiotic-resistant strains, were shown.

The role of the airborne transmission in dissemination of infections caused by these pathogens in groups exposed in occupational environments has been empirically confirmed. However, there is still a need to extend the knowledge on the significance of this route in *S. aureus* infection spreading in humans, particularly in the general population.

Modern methods of identification, especially the MALDI-TOF MS, create the possibility to fastly analyse the origin of strains isolated from the air and to confirm their source. The investigations of the tracts of the MRSA transmission in the air (LA-MRSA, HA-MRSA, and LA-MRSA) need to be carried on a much wider scale than currently to prevent the transfer of resistance genes and infections caused by these strains in human.
